# The Transcription Factor Aabzip9 Positively Regulates the Biosynthesis of Artemisinin in *Artemisia annua*

**DOI:** 10.3389/fpls.2019.01294

**Published:** 2019-11-07

**Authors:** Qian Shen, Huayi Huang, Yu Zhao, Lihui Xie, Qian He, Yijun Zhong, Yuting Wang, Yuliang Wang, Kexuan Tang

**Affiliations:** Key Laboratory of Urban Agriculture (South) Ministry of Agriculture, Plant Biotechnology Research Center, Fudan-SJTU-Nottingham Plant Biotechnology R&D Center, School of Agriculture and Biology, Shanghai Jiao Tong University, Shanghai, China

**Keywords:** *Artemisia annua*, artemisinin, bZIP transcription factors, gene expression, metabolic regulation

## Abstract

Artemisinin-based therapies are the only effective treatment for malaria, which reached to 219 million cases and killed 435,000 people in 2017. To meet the growing demand for artemisinin and make it accessible to the poorest, genetic engineering of *Artemisia annua* becomes one of the most promising approaches to improve artemisinin yield. In this work, AabZIP9 transcription factor has been identified and characterized. The expression profile of *AabZIP9* revealed that it was clustered with the artemisinin specific biosynthetic pathway genes *ADS*, *CYP71AV1*, *DBR2*, and *ALDH1*. Furthermore, the transiently dual-LUC analysis showed that the activation of *ADS* promoter was enhanced by AabZIP9. Meanwhile, yeast one-hybrid assay showed that AabZIP9 was able to bind to the “ACGT” *cis*-element present in both *ADS* and *CYP71AV1* promoters. AabZIP9 gene was driven by the constitutive CaMV35S promoter and the glandular trichome specific CYP71AV1 promoter and stably transformed into *A. annua* plants. The transcript level of AabZIP9 was increased in both of the 35S and CYP71AV1 driven transgenic plants compared with the wild type or GUS control plants. All the transgenic *A. annua* plants overexpressing *AabZIP9* showed elevated transcript level of *ADS*, but the transcription levels of *CYP71AV1*, *DBR2*, and *ALDH1* have no significant change in both types of transgenic plants. The significantly upregulated *ADS* promoted the accumulation of artemisinin, dihydroartemisinic acid, and artemisinic acid biosynthesis in the transgenic *A. annua* plants. These results suggest that AabZIP9 can positively regulate the biosynthesis of artemisinin.

## Introduction

For decades, malaria has been a pandemic disease, threatening people in tropical and subtropical regions of the world, compromising millions of lives. The WHO (World Health Organization) reported 219 million cases of malaria in 2017 and 435,000 deaths (WHO, 2018). Artemisinin, a secondary metabolite produced in the wild plant *Artemisia annua*, along with its derivatives is the main ingredient of artemisinin combination therapies (ACTs), which is currently the only effective cure for malaria (Okell et al., 2014). The discovery of artemisinin by Prof. Youyou Tu, who was further awarded the Nobel Prize in Physiology and Medicine in 2015, could significantly elevate the current antimalaria therapies and save millions of lives. In addition to antimalarial activity and due to its diverse promising functions, including anticancer (Tin et al., 2012), viral (Obeid et al., 2013), inflammatory (Chadwick et al., 2013), diabetic (Li et al., 2017), and tuberculosis therapies (Zheng et al., 2017), the demand for artemisinin has risen in the recent years.

Many studies related to artemisinin production have been carried out in various areas in order to elucidate the biosynthetic production of artemisinin through the regulation of its multi enzymatic biosynthetic pathway. The enzymatic conversion of farnesyl pyrophosphate (FPP) into amorpha-4, 11-diene by amorpha diene synthase (ADS) is the first committed step of artemisinin specific biosynthetic pathway (Bouwmeester et al., 1999), which is followed by oxidization of amorpha-4,11-diene to artemisinic alcohol and subsequently to the artemisinic aldehyde by the cytochrome P450 monooxygenase CYP71AV1 (Teoh et al., 2006). The artemisinic aldehyde is then further oxidized to artemisinic acid by CYP71AV1 or alternatively, is reduced to dihydroartemisinic aldehyde by the double bond reductase 2 (DBR2) (Zhang et al., 2008). The later stage of the pathway is then mediated by aldehyde dehydrogenase 1 (ALDH1) that will oxidize dihydroartemisinic aldehyde into dihydroartemisinic acid (DHAA), which is the direct precursor of artemisinin (Teoh et al., 2009). The final stage of artemisinin production is considered to be a photosensitized related, nonenzymatic oxidation reaction (Brown and Sy, 2007; Czechowski et al., 2016).

Despite the fact that artemisinin biosynthetic pathway is relatively well elucidated, few researches and efforts have been carried out regarding the genetic regulation of the pathway. Recently several transcription factors including AaWRKY1 (Ma et al., 2009; Han et al., 2014), AabHLH1 (Ji et al., 2014), AabZIP1 (Zhang et al., 2015), AaMYC2 (Shen et al., 2016), AaNAC1 (Lv et al., 2016), AaMYB1 (Matías-Hernández et al., 2017), AaGSW1 (Chen et al., 2017) as well as various members of AP2/ERF transcription factors (TFs) including, AaERF1, AaERF2, AaORA, and AaTAR1 (Yu et al., 2012; Lu et al., 2013; Tan et al., 2015) have been reported which significantly participate in and regulate various stages of artemisinin biosynthetic pathway. Judd et al., reported that artemisinin is also produced in the nonglandular cells of self-pollinated inbred *A. annua* plants, which means that the regulation of artemisinin biosynthesis may not be limited to only glandular trichomes (Judd et al., 2019). The bZIP transcription factor family is one of the biggest families in plants, which plays important roles in plant growth and development. A variety of important bZIP genes were identified in model plants such as Arabidopsis and rice. To date, only two bZIP transcription factors (TF) have been identified in *A. annua* which regulate the biosynthesis of artemisinin. (Zhang et al., 2015; Zhong et al., 2018). The function of this important TF family in artemisinin biosynthesis remains obscure and needs to be addressed and characterized to enable a full understanding of bZIP functions, especially in regulating plant secondary metabolism.

In this study, we aimed to identify bZIP TFs involved in artemisinin biosynthesis in *A. annua*. A total of 86 putative AabZIP TFs genes were retrieved for coexpression analysis. One candidate bZIP TF, designated as AabZIP9, was highly expressed in trichome-enriched tissues (young-leaf, bud, and flower) and grouped with the artemisinin specific biosynthetic pathway genes.

## Materials and Methods

### Plant Materials

The seeds of *A. annua* (cultivar “Huhao 1”) were bred by our lab for several years in Shanghai, China. The seeds were sown in pots and grown in a growth chamber under standard conditions of light, temperature, and humidity (16/8 light/dark period, 25 ± 2°C, 50%–70% relative humidity). The *Nicotiana benthamiana* (a close relative of tobacco) seeds were also sown in pots and grown under the same conditions as for *A. annua*.

### Bioinformatics Analysis and Gene Isolation

The transcriptome sequences of seven different tissues of *A. annua* were generated by our laboratory (Shen et al., 2018) and translated into protein sequences in six frames. The AabZIP homologs were identified by the HMM search against the *A. annua* protein sequence database with *E* value < 1×10^-5^ using conserved bZIP domain (PF00170) which was obtained from the Pfam database (Punta et al., 2012). The reads of each bZIP TFs in seven different tissues (young leaf, old leaf, flower, bud, root, stem, and seed) were obtained by BLASTN search against each database with *E* value < 1 × 10^-6^, and read counts were normalized by calculating the value of reads per kilobase per million (RPKM). To predict potential AabZIPs transcription factors that could be involved in the biosynthesis of artemisinin, coexpression analysis was performed based on RNA-seq data, using Multi-Experiment Viewer (MeV4.9.0) software (Saeed et al., 2003). Sample clustering was carried out using the HCL method and the evolutionary distances were computed with the Poisson correction and single linkage clustering (Eisen et al., 1998). The bZIP protein sequences of the Arabidopsis (retrieved from TAIR, The Arabidopsis Information Resource) and *A. annua* were aligned with ClustalW (Larkin et al., 2007). The unrooted phylogenetic tree was generated with MEGA6.1 software (Tamura et al., 2013), according to the neighbor-joining method and bootstrapping with 10,000 replicates to evaluate the accuracy of phylogenetic construction. The evolutionary distances were computed with the Poisson correction and partial deletion option method. Nuclear localization signal of AabZIP9 was predicted *via* NucPred online analysis tool (http://nucpred.bioinfo.se/nucpred/) (Brameier et al., 2007).

For gene isolation, the gene-specific primers, AabZIP9-F, and AabZIP9-R, were designated according to the assembled RNA-seq data. PCR was performed according to the manufacturer’s instructions for KOD DNA polymerase (Toyobo, Japan), using *A. annua* young leaf cDNA as template. The PCR products were cloned into the pLB vector (Tiangen, China) and sequenced (Biosune, China). The primers used in this study are listed in **Supplemental Table 2**.

### Generation of Plasmid Vectors

In order to obtain plant overexpression vector and perform dual Luciferase reporter gene assay (dual-LUC), AabZIP9 ORF was amplified with primers P3+P4, and cloned into pHB vector (driven by CaM35S), and this vector was also used for *A. annua* stable transformation. The promoters of *ADS*, *CYP71AV1*, *DBR2*, and *ALDH1*, previously generated by our lab (Hao et al., 2017), were also cloned into pGreenII 0800-LUC reporter plasmid (Hellens et al., 2005). For subcellular localization analysis, *AabZIP9* was amplified with primers P5+P6 and cloned into Gateway vector pENTR-TOPO (Invitrogen, USA) and subsequently recombined into pEarleyGate104 (Earley et al., 2006) by LR reaction (Invitrogen, USA) to generate pEarleyGate104-YFP-AabZIP9. For gene overexpression in *A. annua* plant, the *AabZIP9* was amplified with primers P7+P8, and cloned into a pHB-proCYP vector (BamHI/XbaI, the CaM35S promoter was replaced by the trichome specific *CYP71AV1* promoter) *via* ClonExpress^®^ II One Step Cloning (Vazyme, China). For yeast one-hybrid assay, the open reading frames (ORFs) of *AabZIP9* was amplified with primers P9+P10 and then cloned into pB42AD (EcoRI/XhoI) by using ClonExpress II (Vazyme, China). The artificial synthesized triplicate *cis*-element promoter segments contain possible bZIP binding element from *ADS*, *CYP71AV1*, *DBR2*, and *ALDH1* were inserted into pLacZ (EcoRI/XhoI) to generate reporter vector. The sequences of artificially synthesized triplicate *cis*-elements were listed in **Supplemental Table 2**.

### Subcellular Localization Analysis and Dual-LUC Assay via *N. benthamiana* Leaf Infiltrations

The leaf infiltration assay was carried out as described by Moses et al. with minor modifications (Moses et al., 2015). Briefly, the plant expression constructs for expression in *N. benthamiana* were transformed into *Agrobacterium tumefaciens* strain GV3101. For dual-LUC analysis, the four pGreenII 0800-LUC reporter plasmids were each cotransformed with the helper plasmid pSoup19 into GV3101. All strains were first incubated overnight (180 rpm) at 28°C in 5 ml LB broth medium, supplemented with antibiotics (50 mg/L rifampicin, 50 mg/L kanamycin, and 20 mg/L gentamycin) and containing 10-mM MES (pH 5.7) and 40-µM acetosyringone. All the *Agrobacterium* cultures were collected by centrifugation (at 4,000 g), and then resuspended in 5 ml of infiltration buffer (200-µM acetosyringone, 10-mM MgCl2, and 10-mM MES, pH 5.7) to reach the OD600 = 0.6 and then incubated at room temperature for 3 h. For subcellular localization analysis, the strains were infiltrated into fully expanded 4-week-old *N. benthamiana* leaves. After 3 days, a piece of infiltrated leaf was cut and the images were obtained under LAS AF Lite laser scanning confocal microscopy (Leica, Germany), with argon laser excitation at 488 nm and a 505–550-nm emission filter set. Subcellular localization was done in three biological replicates. For dual-LUC analysis, the reporter strains and effector strain were mixed at the ratio 1:3, then the bacteria mixtures were infiltrated into fully expanded 4-week-old *N. benthamiana* leaves. The pHB-GFP was used as negative control. After 48 h, the infiltrated leaf discs (1 cm in diameter) were collected for dual-LUC assay using dual-LUC reaction reagents according to the manufacturer (Promega, USA). Four biological replicates were measured for each sample.

### Yeast One-Hybrid Assay

The yeast one-hybrid assay was performed as described previously (Yan et al., 2017). The possible *cis*-elements in the promoters of *ADS*, *CYP71AV1*, *DBR2*, and *ALDH1* were predicted *via* PlantCARE online tools (bioinformatics.psb.ugent.be/webtools/plantcare/html/). In brief, the pB42AD-AabZIP9 plasmid was cotransformed into yeast strain EGY48a with each pLacZ-Boxes, respectively, *via* LiAc mediated method. The cotransformed of pB42AD-AabZIP9 and pLacZ-control was set as negative control. The transformants were cultivated on synthetic minimal double dropout medium deficient in Trp and Ura (SD/-Leu/-Ura) at 30°C for 3 days. Yeast cells grown on SD/-Leu/-Ura medium plates were transferred and suspended with sterile water, then dropped on a selected medium SD-GAL/RAF with X-Gal plate at 30°C for 24–36 h. Six biological replicates were measured for each yeast transformant.

### Plant Transformation of *A. annua*

The plant overexpression construct (pHB-AabZIP9, pHB-pCYP-AabZIP9, and pHB-GUS) were introduced into *Agrobacterium tumefaciens* strain EHA105, and the resulting strains were used in the transformation of *A. annua*. The transgenic plants of *A. annua* were generated as described previously (Shen et al., 2012). The rooted transformed *A. annua* plantlets were transferred into soil pots in the growth chamber for one month and then transplanted into the greenhouse. The T_0_ transgenic plants of *A. annua* were confirmed by genomic DNA-based PCR on both AabZIP9 and Hyg (Hygromycin) resistant genes by using primers P11+P12, P13+P14, and Hyg-F+ Hyg-R, respectively. All the primers used in genomic DNA-based PCR were listed in **Supplemental Table 2**.

### Relative Expression Analysis via Quantitative Real-Time PCR

Leaf samples were collected as described previously (Yan et al., 2017). Leaf1 to leaf5 (**Figure 5A**) were collected for gene expression analysis. Samples were picked and immediately frozen in liquid nitrogen and stored at –80°C. The total RNA was extracted by RNAprep pure Plant Kit according to the manufacturer (Tiangen, China). 1.0 µg of RNA was used to synthesize the cDNA using PrimeScript RT Master Mix Kit (TaKaRa, China). qRT-PCR assays were performed by using SuperReal PreMix SYBR Green Kit (Tiangen, China) on the LightCycle96 machine (Roche, Switzerland) as reported before (Shen et al., 2016). The relative expression levels of genes were compared with the expression of *A. annua β*-actin and calculated by the 2^-ΔCt^. Three biological×four technical replicates were measured for each sample. All the primers used in qRT-PCR were listed in **Supplemental Table 2**.

### HPLC Analysis of Metabolites in *A. annua*

Four months old plant leaves were collected for HPLC analysis, samples of the transgenic plants and control plants were prepared as described previously (Lu et al., 2013). Leaf samples were dried in a drying oven at 45°C and then ground to powder and extracted with methanol in an ultrasonic processor under the conditions of 25°C and 50W for 30 min. The samples were centrifuged for 10 min at 10,000 g and then the supernatants were passed through a 0.25-µm membrane.

The filtrates were then used for metabolites analysis by Waters Alliance 2695 HPLC system coupled with a Waters 2420 ELSD detector. The conditions for HPLC were set as described previously (Lu et al., 2013). Standard of artemisinin was purchased from Sigma and standard of dihydroartemisinic acid and artemisinic acid were bought from Guangzhou Honsea Sunshine Bio Science and Technology Co. Ltd (Honsea Sunshine Bio, China). Three biological replicates were measured for each sample.

## Results

### Global Expression Analysis of bZIP Transcription Factors in *A. annua*

**Figure 1:**
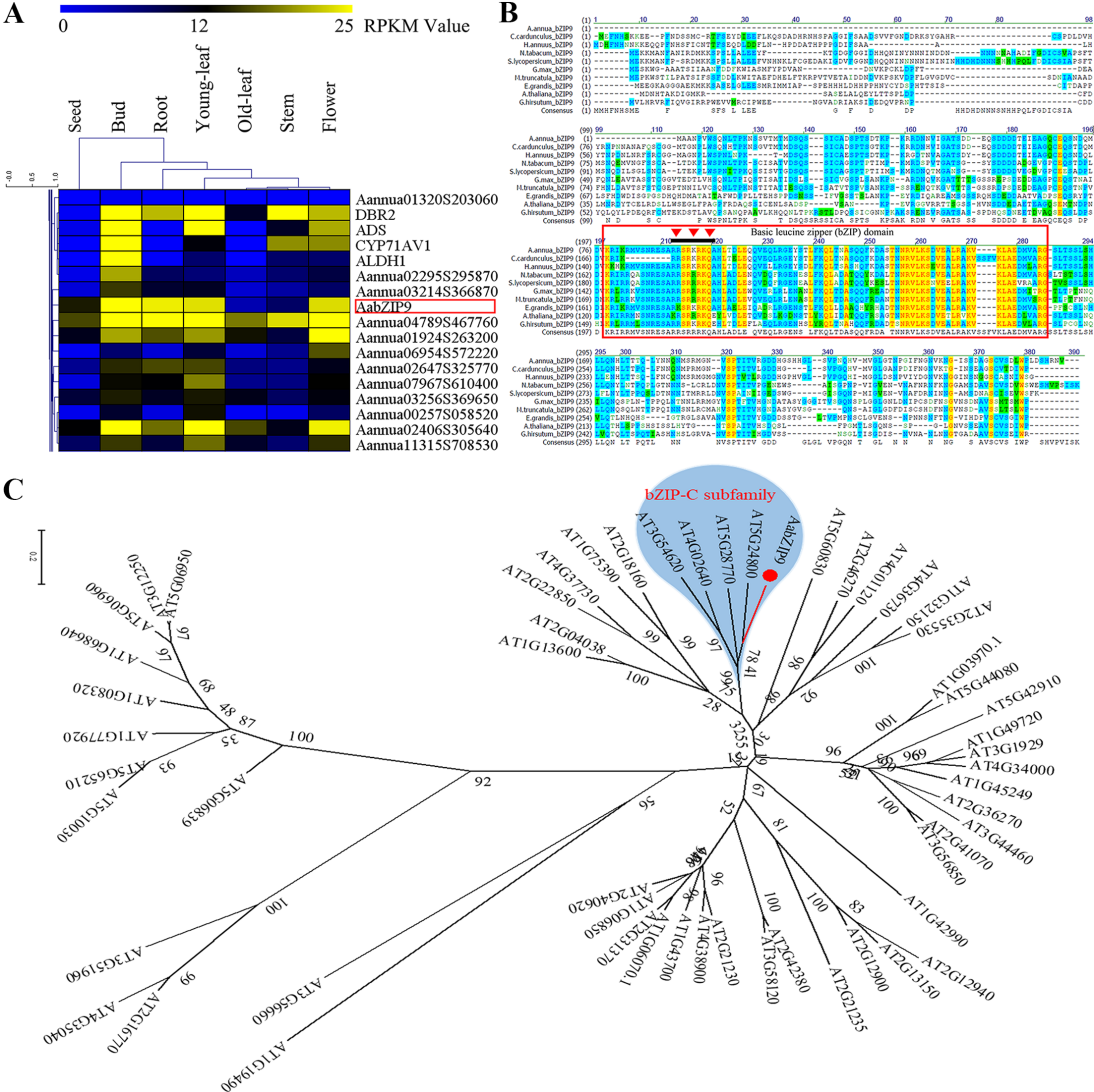
Global expression profile, sequence alignment, and phylogenetic analysis of AabZIP9 transcription factor in *A. annua*. **(A)** Hierarchical cluster analysis of bZIP TFs (partial) in *A. annua*. The color scale at the top represents the value of RPKM (Reads per Kilobase per Million mapped reads); **(B)** Alignment of AabZIP9 with related bZIP proteins from *Helianthus annuus*, *Nicotiana tabacum*, *Medicago truncatula*, *Arabidopsis thaliana*, *Solanum lycopersicum*, etc. The amino acid sequence of AabZIP9 was aligned with *C.cardunculus* bZIP9 (KVH91346.1), *H.annuus* bZIP9 (XP_022029119.1), *N.tabacum* bZIP9 (XP_016449896.1), *M.truncatula* bZIP9 (XP_003588595.1), *G.max* bZIP9 (XP_006601038.1), *S.lycopersicum* bZIP9 (XP_004244469.1), *E.grandis* bZIP9 (XP_010049789.1), *G.hirsutum* bZIP9 (XP_016720593.1), and *A. thaliana* bZIP9 (NP_568457.1) using the Vector NTI 10.3 software. The amino acid residues shaded with yellow, blue, and green, respectively, the high, intermediate and low sequence similarity of AabZIP9 with respect to the other species. The conserved bZIP domain region was marked with red box, and the nuclear localization signal of AabZIP9 was marked with red inverted triangle. **(C)** Phylogenetic analysis of AabZIP9 and all the bZIP TFs from Arabidopsis. The amino acid sequences of Arabidopsis bZIP TFs were obtained from the Arabidopsis Information Resource database (http://www.arabidopsis.org). The phylogenetic tree presented here was analyzed by MEGA 6.1 software, according to the neighbor-joining method. The type-c bZIP subfamily was highlighted by blue background.

The transcriptomic sequencing data of seven different tissues of *A. annua* was generated *via* RNA-seq in our lab (Shen et al., 2018). The genes encoding putative bZIP proteins in *A. annua* were identified using conserved bZIP domain (PF00170) through the HMM (hidden Markov model) search program providing the 86 unique genes encoding proteins containing the bZIP domain (**Supplementary Table 1**). Since the biosynthesis of plant secondary metabolites is usually species or tissue specific (Goossens, 2014), to identify transcription factors involved in the regulation of artemisinin biosynthesis we looked at their expression across tissues. We identified several TFs of the bZIP family which showed expression profiles similar to that of genes of the artemisinin biosynthetic pathway such as *ADS*, *CYP71AV1*, *DBR2*, and *ALDH1* (**Figure 1A** and **Supplementary Figure 1**). *AabZIP9* is one of the candidates that clustered with those pathway genes bearing a higher expression level in buds and young leaves, which could be associated with its involvement in artemisinin biosynthesis.

### Gene Cloning and Identification

The full length of *AabZIP9* (GenBank accession: MG584701) was found to be a 762 bp open reading frame, coding 254 amino acids, isolated from the cDNA of young leaves from *A. annua* “Huhao 1” cultivar. The BLAST results of bZIP9 proteins from other plants species are shown in **Figure1B**. The results indicate that AabZIP9 has 83% of protein sequence identity with *Cynara cardunculus* CabZIP9, 80% with *Helianthus annuus* HabZIP9, 62% with *Nicotiana tabacum* NtZIP9, 55% with *Solanum lycopersicum* SlbZIP9, and 52% with *Arabidopsis thaliana* AtbZIP9. The analysis of the AabZIP9 protein sequence performed with the NucPred online program (Brameier et al., 2007) revealed the presence of a putative nuclear localization signal (NLS; prediction score = 0.92.) indicated with an inverted red triangle in **Figure 1B**. Arabidopsis bZIP TFs (AtbZIP) are subdivided into ten groups accordingly to the similarity of their core domain and additional conserved motifs (Jakoby et al., 2002). Based upon sequence similarity, AabZIP9 groups together with type-C AtbZIPs which includes AtbZIP9 (At5g24800), AtbZIP10 (At4g02640), AtbZIP25 (At3g54620), and AtbZIP63 (At5g28770) genes (**Figure 1C**).

### AabZIP9 Localizes to the Nucleus Where it Activates the Expression of the *ADS* Gene

**Figure 2:**
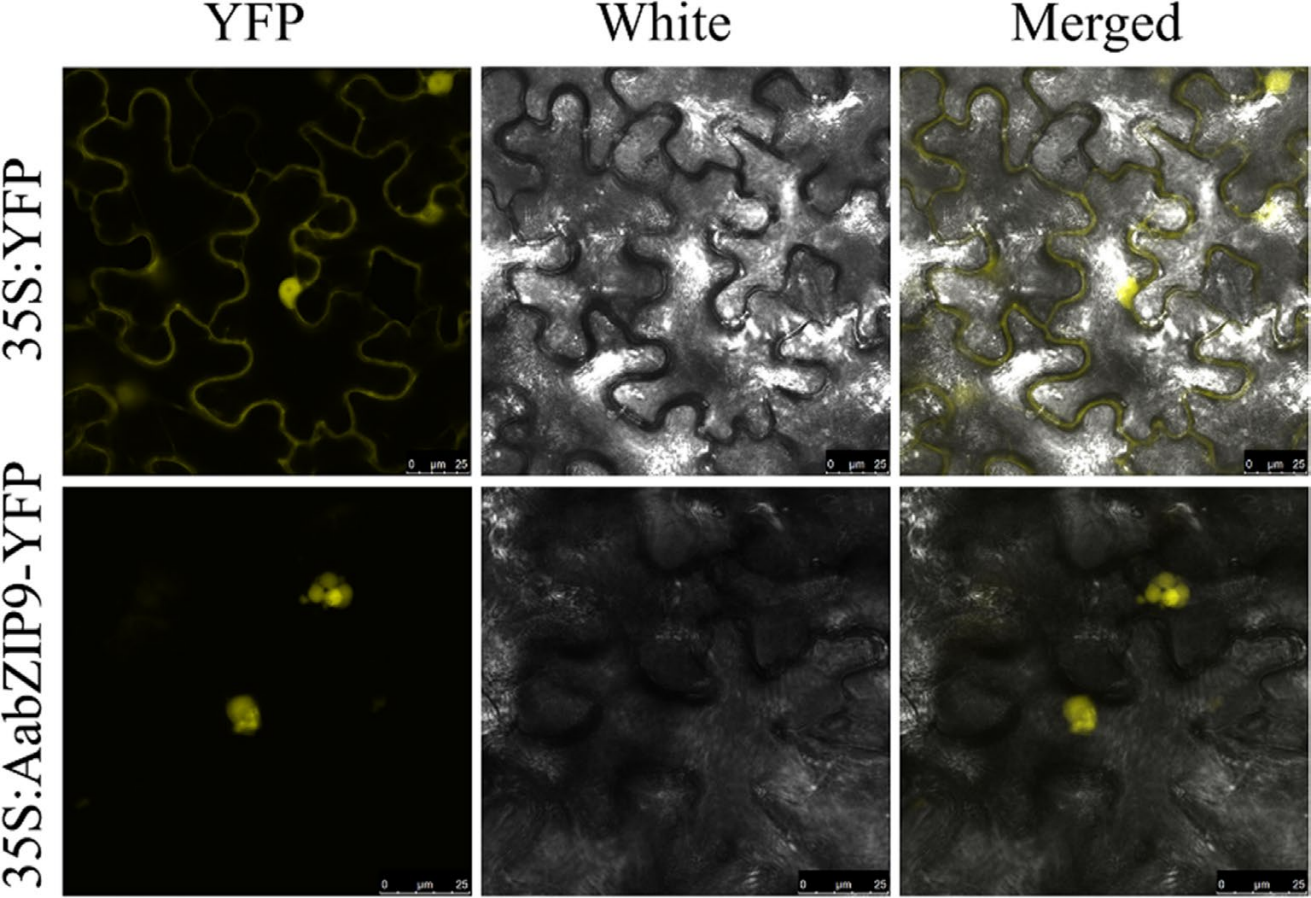
Nuclear localization of AabZIP9 proteins in *N. benthamian*. The coding sequence of AabZIP9 was in-frame fused with YFP, and under the control of the 35S promoter, then transferred into *Agrobacterium* GV3101 and infiltrated into leaves of *N. benthamiana*. The *Agrobacterium* strain harboring empty vector was used as control. Fluorescence (left), bright field (middle), and the corresponding merged (right) images of cells were observed under LAS AF Lite laser scanning confocal microscopy (Leica, Germany).

The subcellular localization of AabZIP9 was examined *via* transient expression in epidermal cell of *N. benthamiana* of the *AabZIP9* gene fused with the YFP reporter (35S:AabZIP9-YFP). The fusion protein (35S:AabZIP9-YFP) showed strong YFP fluorescence in the nucleus, whereas the control YFP (35S:YFP) signal was distributed in both the cytoplasm and the nucleus (**Figure 2**). This confirms that AabZIP9 localizes to the nucleus.

**Figure 3:**
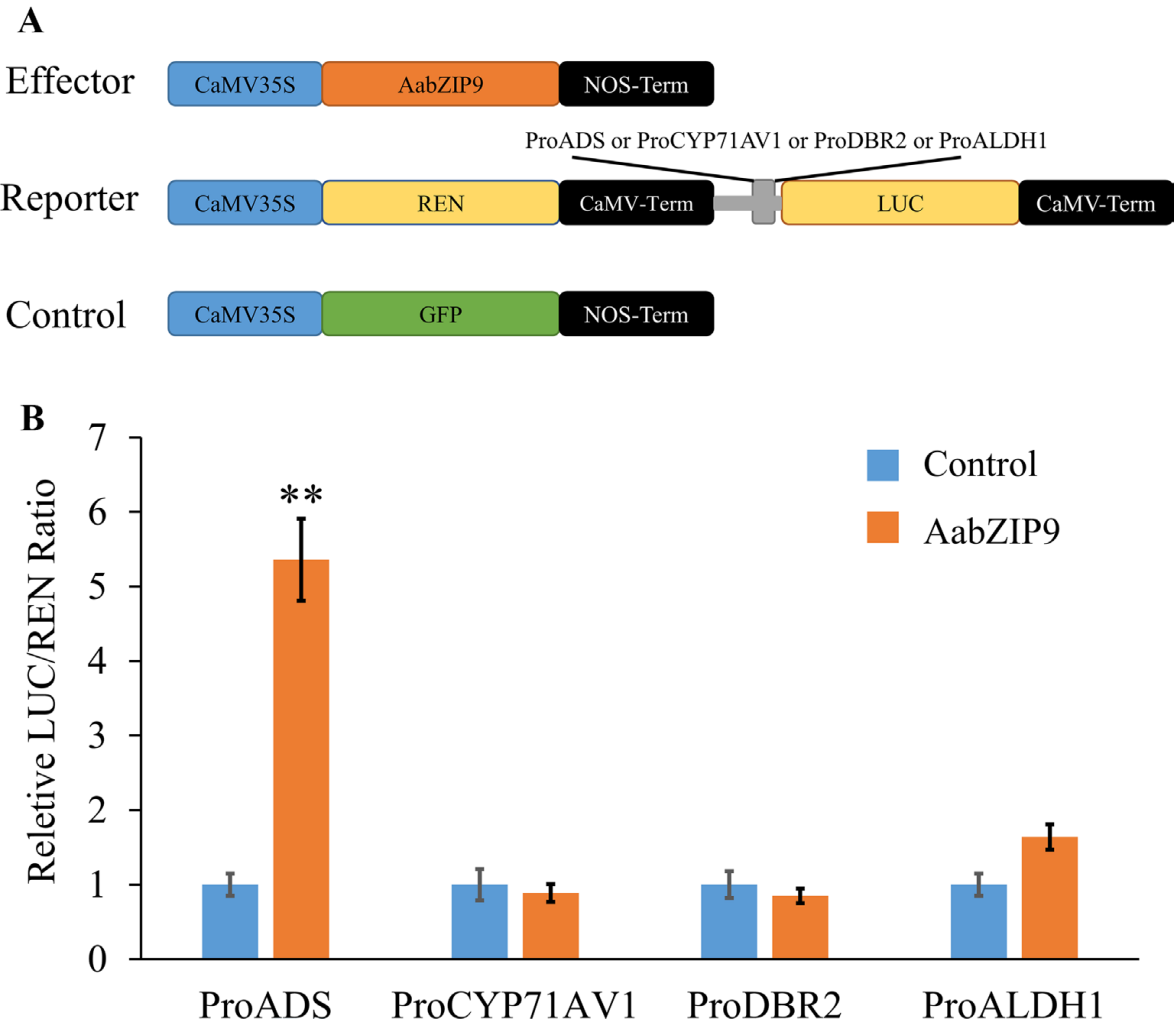
Transient dual-LUC analysis of AabZIP9 with four artemisinin biosynthesis specific pathway gene promoters. **(A)** Schematic representation of effector and reporter constructs used in the dual-LUC assays. Effector construct contains the AabZIP9 coding sequence driven by the CaMV35S promoter. ProADS : LUC, ProCYP71AV1:LUC, ProDBR2:LUC, and ProALDH1:LUC were used as reporter constructs. The REN (*Renilla reniformis*) was driven by the CaMV35S promoter and used as internal control. The effector construct contains green fluorescent protein (GFP) driven by CaMV35S promoter was used as negative control. **(B)** Dual-LUC analysis of AabZIP9 activating of amorpha diene synthase (*ADS*) promoter in *N. benthamiana* leaves. The values were reached by calculating the relative ratio of LUC activities to REN activities (LUC/REN), and the negative control was set to one. Error bars represent ± SD (n = 4), *t-test*, **P < 0.01).

To determine the activation specificity of AabZIP9, a dual-luciferase (dual-LUC) assay was performed in *N. benthamiana* leaves. The dual-LUC assay system provides an efficient means of performing two reporter assays. In this assay, the firefly and renilla luciferases are simultaneously expressed and measured sequentially from a single sample to improve experimental accuracy. The full-length cDNA of *AabZIP9* was inserted into the plant overexpression vector pHB as an effector construct (pHB-AabZIP9) (**Figure 3A**). The four constructs containing promoters of genes *ADS*, *CYP71AV1*, *DBR2*, and *ALDH1* in the pGreenII 0800-LUC vector were previously generated by our lab and used as reporter constructs. The effector and reporter constructs were transiently expressed in *N. benthamiana* leaves using *Agrobacterium tumefaciens*-mediated coinfiltration. The pHB-GFP construct containing the green fluorescent protein (GFP) reporter was also coinfiltrated with each constructs as a negative control. The dual-LUC assay showed that only the *ADS* promoter’s activity was significantly enhanced by AabZIP9 transcription factor when compared with the GFP control (5.1-fold, **P <0.01), whereas the activity of *CYP71AV1*, *DBR2*, and *ALDH1* promoters exhibited nonsignificantly differences (**Figure 3B**).

### AabZIP9 Bind to “ACGT” *Cis*-Elements Within the Promoters of *ADS* and *CYP71AV1*

**Figure 4:**
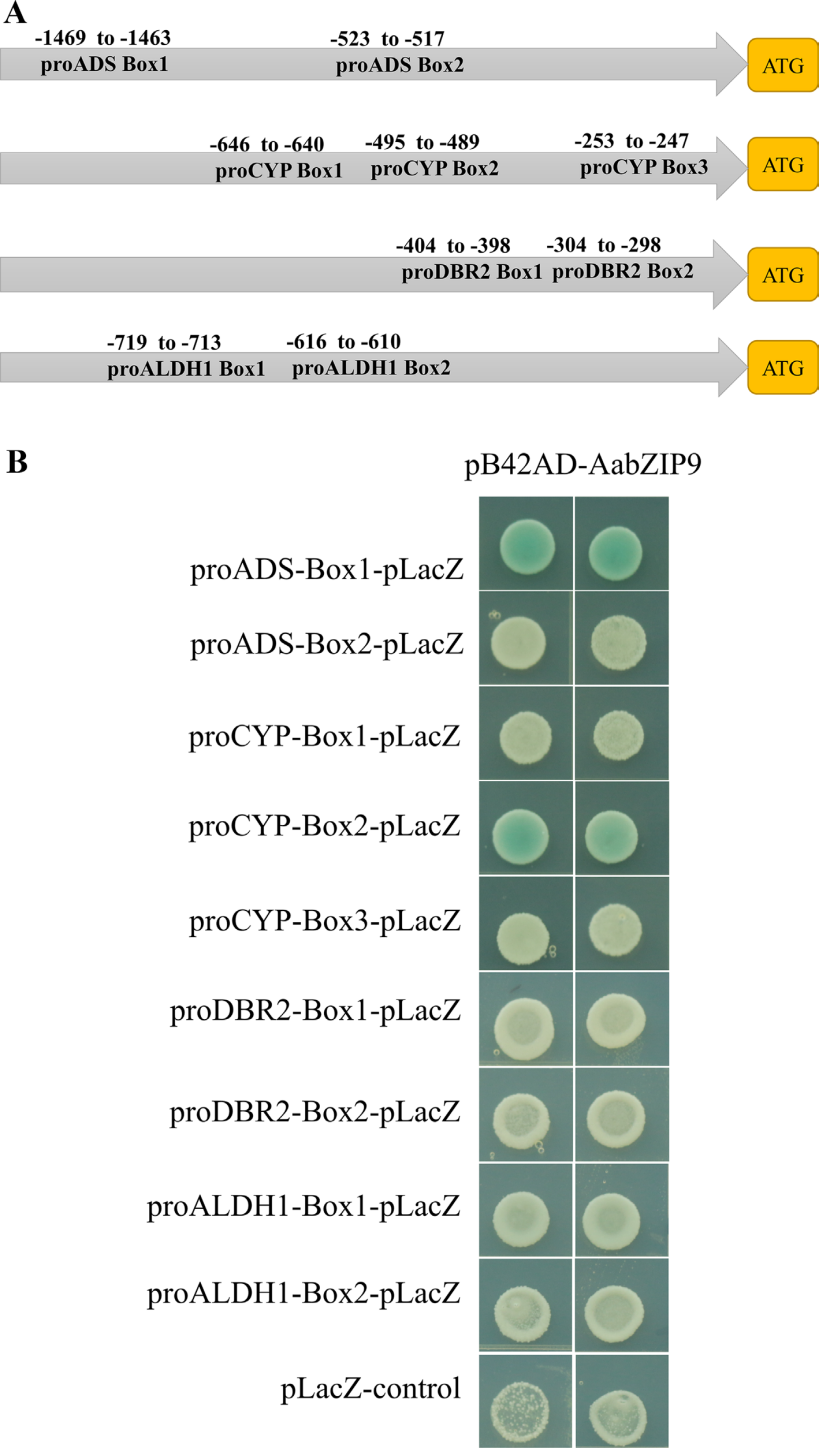
Binding assay of AabZIP9 to “ACGT” *cis*-elements in the promoter. **(A)** Schematic representation of all “ACGT” *cis*-elements boxes position in the four promoters. **(B)** The result of yeast one-hybrid assay for the interaction between AabZIP9 with DNA motifs. Triple of each “ACGT” *cis*-elements with flanking sequences were used as bait, respectively. The transformants were cultivated on selected medium SD-GAL/RAF with X-Gal at 30°C for 24–36 h.

Transcription factors have DNA binding domain for binding to the promoter of corresponding genes, thereby inducing or repressing transcription of downstream target genes. Several studies reported that plant bZIP proteins preferentially bind to DNA sequences with an “ACGT” core in the A-box (TACGTA), C-box (GACGTC), and G-box (CACGTG) (Jakoby et al., 2002). Therefore, we performed promoter *cis*-elements analysis in PlantCARE (Lescot et al., 2002), to identify the putative binding site of bZIP proteins in the promoter region of genes of artemisinin biosynthetic pathway. Sequence analysis revealed that the promoters of *ADS*, *CYP71AV1*, *DBR2*, and *ALDH1* contained two, three, two, and two putative bZIP binding *cis*-elements, respectively (**Figure 4A**). To determine whether AabZIP9 binds to the *cis*-elements of the *ADS*, *CYP71AV1*, *DBR2*, and *ALDH1* promoters *in vivo*, a yeast one-hybrid assay was performed. Each of these putative *cis*-elements along with the nucleotides in the flanking region was artificially synthesized into 3 × element triple fragment and inserted into the yeast one-hybrid pLacZ reporter vector (**Supplementary Figure 2**). The ORF of *AabZIP9* was inserted into the pB42AD vector to generate effector construct (**Supplementary Figure 2**). The effector along with each respective reporter construct were cotransferred into the yeast EGY48 strain *via* LiAc mediated method as described before (Yan et al., 2017). As shown in **Figure 4B**, only the yeast cells harboring pB42AD-AabZIP9, proADS-box1, and proCYP71AV1-box1 were able to grow on the SD-Gal-Raf selective medium containing 20-mM X-gal as displayed by the development of the blue color. This indicates that AabZIP9 binds to the “ACGT” *cis*-elements within the promoters of genes *ADS* and *CYP71AV1*, but it fails to bind to the *cis*-elements of the other genes we tested. We think that this is due the flanking sequences of the *cis*-element which may affect the binding ability of AabZIP9 as it has previously been reported (Dombrecht et al., 2007).

### AabZIP9 Regulates the Transcription of Genes in the Artemisinin Biosynthetic Pathway

Since AabZIP9 was able to bind to and activate *ADS* promoter, it was interesting to know whether overexpression of *AabZIP9* could active *ADS* gene and therefore promote artemisinin biosynthesis. Meanwhile, in order to compare the efficiency of constitutive and trichome specific promoters, the promoter of CaMV35S and the promoter of gene *CYP71AV1* were used for overexpressing *AabZIP9* in *A. annua* plants respectively. We constructed the pHB-35S-AabZIP9 and pHB-proCYP-AabZIP9 plant overexpression vector and used pHB-35S-GUSplus vector as control one (**Supplementary Figure 3**). All these vectors were transformed into *A. annua via* the *Agrobacterium*-mediated method as described before (Zhang et al., 2009). In total, eight (driven by 35S promoter) and 10 (driven by the promoter of gene *CYP71AV1*) independent transgenic plants of *A. annua* were confirmed by genomic DNA-based PCR on both AabZIP9 and Hyg (Hygromycin) resistant genes.

**Figure 5:**
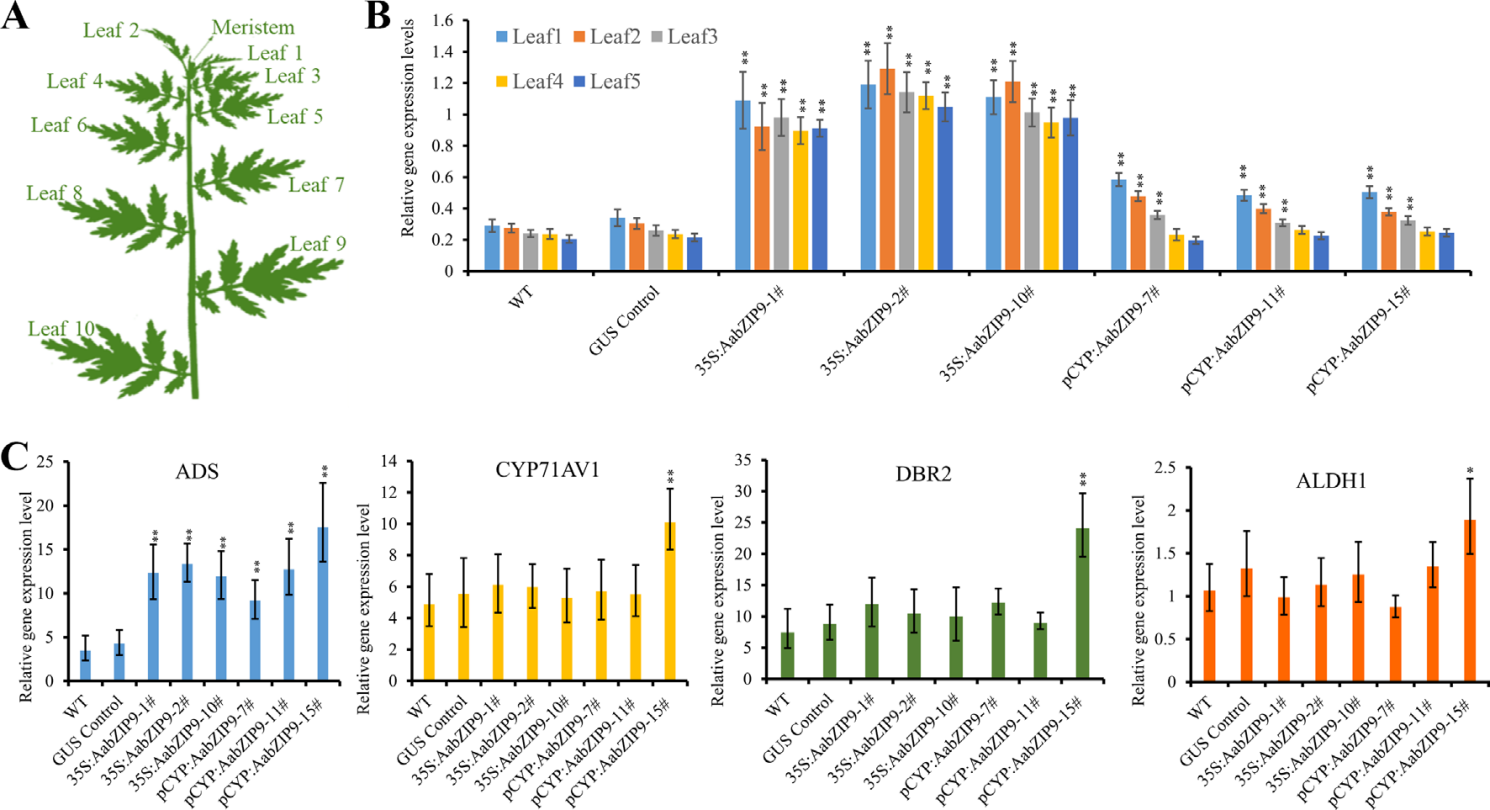
Overexpression of *AabZIP9* upregulated expression levels of amorpha diene synthase (*ADS*) genes. **(A)** Schematic diagram of *A. annua* plant shows different positions of leaves (Yan et al., 2017). **(B)** The expression levels of *AabZIP9* in five different position of leaves in the wild type, GUS control and 35S promoter or CYP71AV1 promoter driven overexpressed plants. *β*-actin was used as the internal standard. Error bars indicate ± SD of four technical replicates. Statistical significance was determined by Student’s *t-test* with paired and two-tailed distribution methods. Asterisks indicate the difference between overexpressing transgenic plants and wild type plants at the same position of each leaf (**, P < 0.01). **(C)** Relative expression of *ADS*, *CYP71AV1*, *DBR2*, and *ALDH1* in leal2 of each wild type, GUS control and 35S promoter or CYP71AV1 promoter driven overexpressed plants. *β*-actin was used as the internal standard. Error bars indicate ± SD of three biological×four technical replicates. Statistical significance was determined by Student’s *t-test* with paired and two-tailed distribution methods. Asterisks indicate the difference between overexpressing transgenic plants and wild type plants (**, P < 0.01; *, P < 0.05)

Quantitative real-time PCR (qRT-PCR) was performed to measure the expression levels of *AabZIP9* in the transgenic plants. Previous research on expression profile of *CYP71AV1* shows that this promoter is highly expressed in young leaves and its expression gradually declines with age (Wang et al., 2012; Yu et al., 2012). Therefore, the expression levels of *AabZIP9* in transgenic and wild-type plants were compared among five different development stages of leaves (from young to old) (**Figure 5A**). As shown in **Figure 5B**, the expression of *AabZIP9* in 35S:: AabZIP9 lines is three to four times higher than in wild type and control plants. However, when the expression of *AabZIP9* was driven by the trichome specific CYP71AV1 promoter, it showed a pattern of expression similar to that of *CYP71AV1* with higher expression in young than in old leaves (Wang et al., 2012; Yu et al., 2012). Nevertheless, the expression levels of *AabZIP9* (in leaf1 and leaf2) in the *CYP71AV1* promoter driven transgenic lines were increased about 1.5- to 2-folds when compared with wild-type or GUS control plants (in leaf1 and leaf2), while the expression levels of *AabZIP1* showed no significant difference between transgenic lines and wild-type or GUS control in the leaves from leaf3 to leaf5, **Figure 5B**.

Taking into account the expression pattern of *AabZIP9* in different leaves, we used the second leaf from each transgenic and wild-type plants to measure the steady state level of genes in the artemisinin biosynthetic pathway. As expected, the expression level of *ADS* gene was upregulated in both 35S and *CYP71AV1* promoters driven transgenic plants. Intriguingly, the expression levels of *ADS* in both 35S and *CYP71AV1* promoters driven transgenic plants were similar, although the *AabZIP9* gene showed higher expression levels in 35S promoter driven transgenic plants than in the *CYP71AV1* promoter driven plants (**Figure 5C**). In most of the transgenic plants, the expression levels of *CYP71AV1*, *DBR2*, and *ALDH1* showed no significant difference between the transgenic and wild-type plants, except in the pCYP : AabZIP9-15 plant (**Figure 5C**). *ADS*, *CYP71AV1*, *DBR2*, and *ALDH1* were all upregulated in plant pCYP : AabZIP9-15 compared to wild-type and control (**Figure 5C**).

### Overexpression of AabZIP9 Increases Artemisinin, Artemisinic Acid and Dihydroartemisinic Acid Biosynthesis in *A. annua* Transgenic Plants

**Figure 6:**
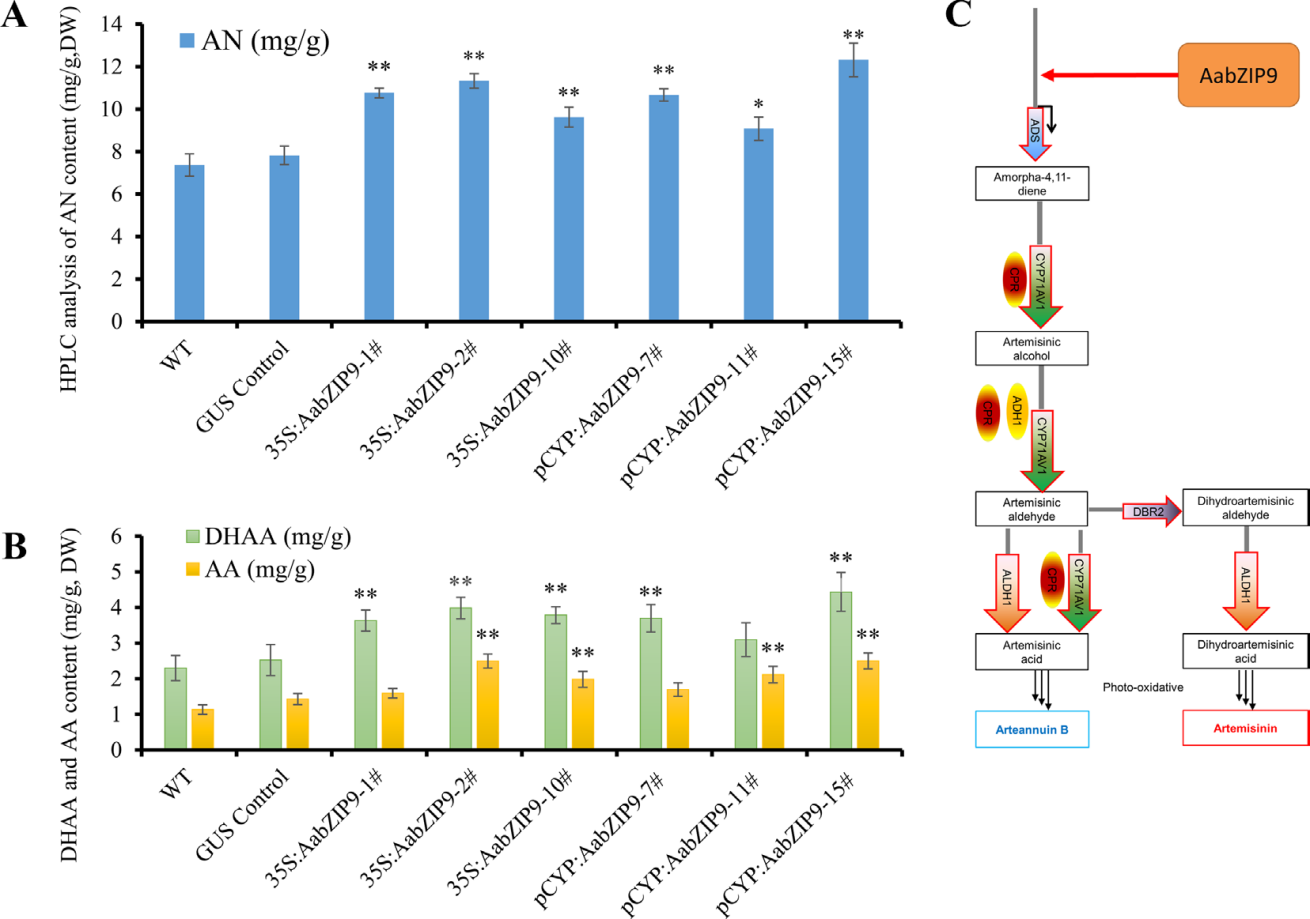
High-performance liquid chromatography (HPLC) analysis of metabolites in transgenic plants overexpressing *AabZIP9*. **(A)** Contents of artemisinin (AN) in the 4-month-old leaves of wild type, GUS control, and 35S promoter or CYP71AV1 promoter driven overexpressed plants, determined by HPLC. The contents of AN in transgenic plant leaves were compared to the wild type plants. Statistical significance was determined by *t-test* (**, P < 0.01; *, P < 0.05). Asterisks indicate the difference between overexpressing transgenic plants and wild type plants. **(B)** Contents of dihydroartemisinic acid (DHAA) and artemisinic acid (AA) in the 4-month-old leaves of wild type, GUS control, and 35S promoter or CYP71AV1 promoter driven overexpressed plants, determined by HPLC. The contents of DHAA and AA in transgenic plant leaves were compared to the wild type plants. Statistical significance was determined by *t-test* (**, P < 0.01; *, P < 0.05). Asterisks indicate the difference between overexpressing transgenic plants and wild type plants. Three biological replicates were measured for each sample. **(C)** A simplified model of action of AabZIP9 in regulating artemisinin biosynthesis in *Artemisia annua*.

Overexpression of *AabZIP9* did not cause any visible morphological modification and all transgenic lines appear similar to the wild-type (**Supplementary Figure 4**). The artemisinin, dihydroartemisinic acid, and artemisinic acid contents in leaves of the 4-month-old *A. annua* plants were analyzed by high-performance liquid chromatography (HPLC). The results of the HPLC analysis revealed that in the 35S::AabZIP9 and CYP::AabZIP9 lines the content of artemisinin, dihydroartemisinic acid, and artemisinic acid level are 23.2%–67.1%, 34.5%–92.8%, and 40.4%–121.2% higher than in wild-type plants (**Figures 6A, B**). These results indicated that AabZIP9 is a positive regulator of artemisinin biosynthesis.

## Discussion

Due to the important medicinal properties of artemisinin, such as antimalarial, anticancer (Tin et al., 2012), antituberculosis (Zheng et al., 2017), understanding how the artemisinin biosynthetic pathway is regulated, is of extreme relevance. Despite the considerable progresses which have been achieved in producing artemisinin in microbes *via* semisynthetic synthesis (Ro et al., 2006; Paddon et al., 2013), *A. annua* is still the main resource for artemisinin (Peplow, 2016). However, the transcriptional regulation of this important pathway in *A. annua* is not yet well established. The bZIP transcription factors are one of the largest transcriptional regulators playing crucial roles in plant physiological processes, plant development, plant metabolism, and in numerous biotic/abiotic stress responses (Ang et al., 1998; Chen et al., 2016) (Osterlund et al., 2000; Tang et al., 2012). To date, the abscisic acid (ABA) response AabZIP1 transcription factor and AaABF3 are the only two bZIP TFs, which have been reported to play a significant role in the regulation of the artemisinin biosynthesis in *A. annua* (Zhang et al., 2015; Zhong et al., 2018). In this study, we isolated and characterized a new bZIP transcription factor, named AabZIP9, which regulates the biosynthesis of artemisinin.

Coexpression analysis has proved to be an efficient tool for successful gene discovery in plant secondary metabolism. Based on the RNA-seq data from different tissues of *A. annua*, 86 putative bZIP TFs in *A. annua* were retrieved and subject to coexpression analysis with the artemisinin specific biosynthetic pathway genes (*ADS*, *CYP71AV1*, *DBR2*, and *ALDH1*) (**Figure 1A**). Sequence alignment and phylogenetic analysis based on the Arabidopsis bZIP TFs classification, revealed that AabZIP9 belongs to the type-C subgroup of bZIP TFs (**Figure 1C**). There are only a few genes of this bZIP C-type subgroup in plants which have been cloned and characterized, including maize *OPAQUE2* and parsley *CPFR2*. The bio-functions of this subgroup in plants are limited, and the information available on *OPAQUE2* indicates that it regulates seed storage protein production (Vicente-Carbajosa et al., 1997), whereas *CPFR2* might be involved in responses to light (Weisshaar et al., 1991). However, our AabZIP9 was found to be involved in plant secondary metabolism and it was of interest to elucidate if AabZIP9 regulates storage protein genes expression in the *A. annua* and responses to light signalling.

The plant bZIP proteins preferentially bind to DNA sequences with an “ACGT” core *cis*-element. The yeast one-hybrid result indicated that AabZIP9 interacts with the “ACGT” elements in the promoter region of the *ADS* and *CYP71AV1* (**Figure 4B**). Furthermore, the *in vivo* transactivation of AabZIP9 to the *ADS* promoter was confirmed by transiently *Agrobacterium* infiltration in tobacco leaf and stably transformed in *A. annua* plants (**Figures 3B** and **5C**). Yeast one-hybrid result showed that AabZIP9 binds to the “ACGT” element in the *CYP71AV1* promoter (**Figure 4B**). However, the results of the dual-LUC assay indicate that AabZIP9 did not affect the activity of *CYP71AV1* promoter (**Figure 3B**), and no significant upregulated gene expression of *CYP71AV1* was found in almost all of the *AabZIP9* overexpressed *A. annua* plants (both 35S and *CYP71AV1* promoter driven transgenic plants) (**Figure 5C**). These results suggest that *ADS* is a target gene of AabZIP9 transcription factor (**Figure 6C**). AabZIP9 was founded only able to promote the expression level of *ADS* gene, which limited its functions in promoting the whole artemisinin biosynthetic pathway and yielded lower artemisinin content compared with our previous ones. An abnormal gene expression pattern was found in the pCYP : AabZIP9-15 transgenic plant as showed in **Figure 5C**. *ADS*, *CYP71AV1*, *DBR2*, and *ALDH1* were all upregulated in plant pCYP : AabZIP9-15, and as a consequence, the highest content of artemisinin was detected in this plant (**Figure 6A**). According to our finding, we speculated that there is a possibility of silencing or even destruction of an artemisinin biosynthesis negative regulator gene during transformation. The identification of possible silenced negative regulator and its characterization may introduce and open a new insight in artemisinin metabolic engineering and its higher production which will remain for further investigation.

Early this year a report had indicated that the nonglandular trichomes cells can also produce trace amounts of artemisinin (Judd et al., 2019), however, the glandular trichomes were still cells that synthesized and stored of artemisinin. Moreover, the artemisinin specific synthetic pathway genes are all glandular trichome specific (Olsson et al., 2009). Therefore, overexpressing exogenous gene in *A. annua* by glandular trichome specific promoter seems more promising than using a constitutive promoter. In this study, we overexpressed the *AabZIP9* gene by using 35S constitutive promoter and the glandular trichome specific *CYP71AV1* promoter, respectively. However, based on gene expression analysis, we found that the 35S promoter driven transgenic plants showed even higher expression levels of *AabZIP9* than the *CYP71AV1* promoter driven transgenic ones, this results were consistent with Han’s study (Han et al., 2014). The downstream target gene (*ADS*) expression levels showed nearly the same abundance in two types of transgenic plants (**Figure 5C**). Then, we checked the GUS control plants which were also driven by the 35S promoter, the GUS staining assays showed that 35S promoter has very strong activities in most of the tissues such as the nonglandular trichomes, the leaf vein, the stomatal cells, and the mesophyll tissue (**Supplementary Figure 5**), but only few glandular trichomes were stained (**Supplementary Figure 5**). Due to the low expression of the 35S promoter in glandular trichome, the 35S promoter driven overexpressed *AabZIP9* demonstrated limited regulation of target genes in *A.annua* (**Figure 5C**). We speculate that the reason for overexpressing exogenous genes *via* 35S promoter could increase the artemisinin content may partially depend on the nonglandular trichome pathway, which was proved by the previous report (Judd et al., 2019). The *CYP71AV1* promoter region (GenBank: FJ870128.1) is glandular trichome specific, and the results obtained by Han et al. show that *CYP71AV1* promoter is more efficient than 35S promoter in application for artemisinin metabolic engineering (Han et al., 2014). These results led us to use *CYP71AV1* promoter for overexpressing *AabZIP9* in *A. annua* plants. Indeed, the *AabZIP9* gene expression levels were upregulated and the artemisinin contents were promoted in the transgenic plants while compared with the wild-type plants (**Figures 5B** and **6A**). However, based on our results, it is too early to state that this glandular trichome-specific *CYP71AV1* promoter was more effective than the constitutive 35S promoter. It has been reported that the *CYP71AV1* gene was dramatically decreased during *A. annua* leaves development (Wang et al., 2012; Yu et al., 2012). Here, we analyzed the *AabZIP9* transcript levels in five different developmental stages of leaves in the *AabZIP9* overexpressed transgenic plants. The expression pattern of *AabZIP9* that was driven by *CYP71AV1* promoter revealed the same pattern as the *CYP71AV1* gene, which was highly expressed in young leaves (leaf1 and leaf2) while lowly expressed in old leaves (leaf3 to leaf5) (**Figure 5B**). The promoters of genes *ADS*, *DBR2*, and *ALDH1* have similar expression pattern to the promoter of gene *CYP71AV1*, so these promoters may not be ideal promoters for the regulation of the metabolism in the glandular trichomes. In spite of this, we believe that glandular trichomes specific promoters still have the great potential and will be powerful tools in plant metabolism regulation. The truncated *ADS* promoter, even in 400-bp length, is still displaying specific expression in *A. thaliana* trichomes and with a higher activity than the original one (Zhu et al., 2014). Moreover, by laser capture microdissection, we generated a RNA-seq database of trichome cells (Shen et al., 2018), it is hence worthwhile to screen, modify and achieve a series of compact trichome specific core promoters that both trichome specific and constitutively high expressing in all stages of leaves.

## Data Availability Statement

All datasets for this study are included in the article/**Supplementary Material**.

## Author Contributions

QS, YLW, and KT conceived and coordinated the study and wrote the manuscript. QS, HH, and YZ performed the gene cloning, vector constructive, and plant transformation work. LX, QH, and YJZ did the gene expression, subcellular localization, luciferase analysis, and Y1H experiments. YTW and YLW helped with the metabolites analysis by HPLC. All authors reviewed the results and approved the final version of the manuscript.

## Funding

This work was supported by the National Natural Science Foundation for Young Scientists of China (Grant No. 31600231), Major National Science and Technology Program of China for Innovative Drug (Grant No. 2017ZX09101002-003-002), Natural Science Foundation of Shanghai (Grant No. 18ZR1420600), and Natural Science Foundation and China Postdoctoral Science Foundation (Grant No. 2016M590356).

## Conflict of Interest

The authors declare that the research was conducted in the absence of any commercial or financial relationships that could be construed as a potential conflict of interest.

## Supplementary Material

Supplementary Table 1 Amino acid sequences of putative bZIP TFs retrieved from A. annua transcriptome database

Supplementary Table 2 Primers used in this investigation

Supplementary Figure 1 Gene co-expression analysis of artemisinin biosynthetic pathway genes with bZIP transcription factor family by MeV 4.9 software. AabZIP9 (marked with red box) gene was clustered with ADS, CYP71AV1 and ALDH1 genes and had a high expression level in the bud, flower, and young leaf, suggesting it may involve in the regulation of artemisinin biosynthesis.

Supplementary Figure 2 Schematic representation of effector and reporter constructs used in the yeast one-hybrid assays. Effector construct contains the AabZIP9 coding sequence driven by the GAL1 promoter while the reporter constructs contain the artificial synthesized triplicate cis-element promoter segments. The empty pLacZ vector was used as control.

Supplementary Figure 3 Schematic of plant overexpression vector. The AabZIP9 was driven by CaM35S or CYP71AV1 promoter, respectively; Hyg (hygromycin phosphotransferase) was used as resistant selection gene for plant transformation.

Supplementary Figure 4 The morphologies of 2-month-old wild-type, GUS control, 35S promoter and CYP71AV1 promoter driven overexpressed transgenic plants, and no morphology changed via transformation process; Bar, 5 cm.

Supplementary Figure 5 Histochemical GUS staining of transgenic A. annua transformed using the 35S: GUS control plasmid. Glandular, nonglandular and stomata structures were pointed out by arrows. (A) 35S promoter has strong activities in most of the leaf tissues (B, C) only a few glandular trichomes get stained by using 35S promoter.
