# Supplementary material for: The Transcription Factor Aabzip9 Positively Regulates the Biosynthesis of Artemisinin in Artemisia annua
Source: Front Plant Sci. 2019 Nov 7;10:1294. doi: 10.3389/fpls.2019.01294 (PMC6855008; doi:10.3389/fpls.2019.01294)
Supplement: Supplementary Table 2 — Primers used in this investigation [file Table_2.docx]

Table S2

Primers used in this investigation

| Primers | Purpose | Primer Sequence (5’-3’) |
| --- | --- | --- |
| AabZIP9-F | Clone | 5’-CTGTCAAGCTTTGTGATATTTGC-3’ |
| AabZIP9-R | Clone | 5’-CAATAAGAGGCTTAGGAGAGGG-3’ |
| P3 | Clone | 5’-GCAAGCTTATGGCCGCAAACCCTGTCTGG-3’ |
| P4  P5 | Clone | 5’-GCTCTAGAAACGTTACGATGAGAATCCAAAGG-3’ |
|  | TOPO-Clone | 5’-CACCATGGCCGCAAACCCTGTCTGG-3’ |
| P6 | TOPO-Clone | 5’-AACGTTACGATGAGAATCCAAAGG-3’ |
| P7 | pHB vector construct | 5’-CTCTCTCTAAGCTTGGATCCATGGCCGCAAACCCTGTCTGG-5’ |
| P8 | pHB vector construct | 5’-GATACGAACGAAAGCTCTAGAAACGTTACGATGAGAATCCAAAGG-3’ |
| P9 | pB42AD vector construct | 5’-GATTATGCCTCTCCCGAATTCATGGCCGCAAACCCTGTCTGG-3’ |
| P10 | pB42AD vector construct | 5’-GAAGTCCAAAGCTTCTCGAGAACGTTACGATGAGAATCCAAAGG-3’ |
| P11 | Genomic-DNA PCR (35S promoter transgenic line) | 5’-CAACAAGTGGAACAACTGCGAGG-3’ |
| P12 | Genomic-DNA PCR (35S promoter transgenic line) | 5’-AGTTGGGTAACGCCAGGGTTTTC-3’ |
| P13 | Genomic-DNA PCR (CYP promoter transgenic line) | 5’-ACCACGTAAGTTTTCCTTTCTTGG-3’ |
| P14 | Genomic-DNA PCR (CYP promoter transgenic line) | 5’-GTTAAAAATGCCGGGATTAGTACC-3’ |
| Hyg-F | Genomic-DNA PCR (transgenic lines) | 5’-GGAAGTGCTTGACATTGGGGAG-3’ |
| Hyg-R | Genomic-DNA PCR (transgenic lines) | 5’-TCCAGAAGAAGATGTTGGCGAC-3’ |
| RT-AabZIP9 F | Real time PCR | 5’-TTTCAAACTGTGGTGGAATGGC-3’ |
| RT-AabZIP9 R | Real time PCR | 5’-CATCACTGGTTGCCCCAATCAC-3’ |
| RT-ADS F | Real time PCR | 5’-AATGGGCAAATGAGGGACAC-3’ |
| RT-ADS R | Real time PCR | 5’-TTTCAAGGCTCGATGAACTATG-3’ |
| RT-CYP F | Real time PCR | 5’-CGAGACTTTAACTGGTGAGATTGT-3’ |
| RT-CYP R | Real time PCR | 5’-CGAAGCGACTGAAATGACTTTACT-3’ |
| RT-DBR2 F | Real time PCR | 5’-GCGGTGGTTACACTAGAGAACTT-3’ |
| RT-DBR2 R | Real time PCR | 5’-ATAATCAAAACTAGAGGAGTGACCC-3’ |
| RT-ALDH1 F | Real time PCR | 5’-TGAGCCTACTCTATTTACAAACG-3’ |
| RT-ALDH1 R | Real time PCR | 5’-TAACAGTTGACCCAAACAGCA-3’ |
| RT-Actin F | Real time PCR | 5’-CCAGGCTGTTCAGTCTCTGTAT-3’ |
| RT-Actin R | Real time PCR | 5’-CGCTCGGTAAGGATCTTCATCA-3’ |
| proADS Box1F | Y1H placZ vector | 5’-aattcTTAGGTCACGTCTTAATTTAGGTCACGTCTTAATTTAGGTCACGTCTTAATc-3’ |
| proADS Box1R | Y1H placZ vector | 5’-tcgagATTAAGACGTGACCTAAATTAAGACGTGACCTAAATTAAGACGTGACCTAAg-3’ |
| proADS Box2F | Y1H placZ vector | 5’-aattcCTATCACACGTTAGAAGCTATCACACGTTAGAAGCTATCACACGTTAGAAGc-3’ |
| proADS Box2R | Y1H placZ vector | 5’- tcgagCTTCTAACGTGTGATAGCTTCTAACGTGTGATAGCTTCTAACGTGTGATAGg-3’ |
| proCYP Box1F | Y1H placZ vector | 5’- aattcCTAACACACGTATAGCACTAACACACGTATAGCACTAACACACGTATAGCAc-3’ |
| proCYP Box1R | Y1H placZ vector | 5’- tcgagTGCTATACGTGTGTTAGTGCTATACGTGTGTTAGTGCTATACGTGTGTTAGg-3’ |
| proCYP Box2F | Y1H placZ vector | 5’-aattcTCTTTTACGTGTCAATTTCTTTTACGTGTCAATTTCTTTTACGTGTCAATTc-3’ |
| proCYP Box2R | Y1H placZ vector | 5’-tcgagAATTGACACGTAAAAGAAATTGACACGTAAAAGAAATTGACACGTAAAAGAg-3’ |
| proCYP Box3F | Y1H placZ vector | 5’-aattcCCAAACCACGTAAGTTTCCAAACCACGTAAGTTTCCAAACCACGTAAGTTTc-3’ |
| proCYP Box3R | Y1H placZ vector | 5’-tcgagAAACTTACGTGGTTTGGAAACTTACGTGGTTTGGAAACTTACGTGGTTTGGg-3’ |
| proDBR2 Box1F | Y1H placZ vector | 5’-aattcAATTAAACGTGAAAAGTAATTAAACGTGAAAAGTAATTAAACGTGAAAAGTc-3’ |
| proDBR2 Box1R | Y1H placZ vector | 5’-tcgagACTTTTCACGTTTAATTACTTTTCACGTTTAATTACTTTTCACGTTTAATTg-3’ |
| proDBR2 Box2F | Y1H placZ vector | 5’-aattcTGTGATACGTGATATTATGTGATACGTGATATTATGTGATACGTGATATTAc-3’ |
| proDBR2 Box2R | Y1H placZ vector | 5’-tcgagTAATATCACGTATCACATAATATCACGTATCACATAATATCACGTATCACAg-3’ |
| proALDH1 Box1F | Y1H placZ vector | 5’-aattcGCACGCCACGTATGTATGCACGCCACGTATGTATGCACGCCACGTATGTATc-3’ |
| proALDH1 Box1R | Y1H placZ vector | 5’-tcgagATACATACGTGGCGTGCATACATACGTGGCGTGCATACATACGTGGCGTGCg-3’ |
| proALDH1 Box2F | Y1H placZ vector | 5’-aattcTTCTATCACGTAACCAATTCTATCACGTAACCAATTCTATCACGTAACCAAc-3’ |
| proALDH1 Box2R | Y1H placZ vector | 5’-tcgagTTGGTTACGTGATAGAATTGGTTACGTGATAGAATTGGTTACGTGATAGAAg-3’ |
|  |  |  |
|  |  |  |
