# Supplementary figures and images for: The Transcription Factor Aabzip9 Positively Regulates the Biosynthesis of Artemisinin in Artemisia annua
Source: Front Plant Sci. 2019 Nov 7;10:1294. doi: 10.3389/fpls.2019.01294 (PMC6855008; doi:10.3389/fpls.2019.01294)

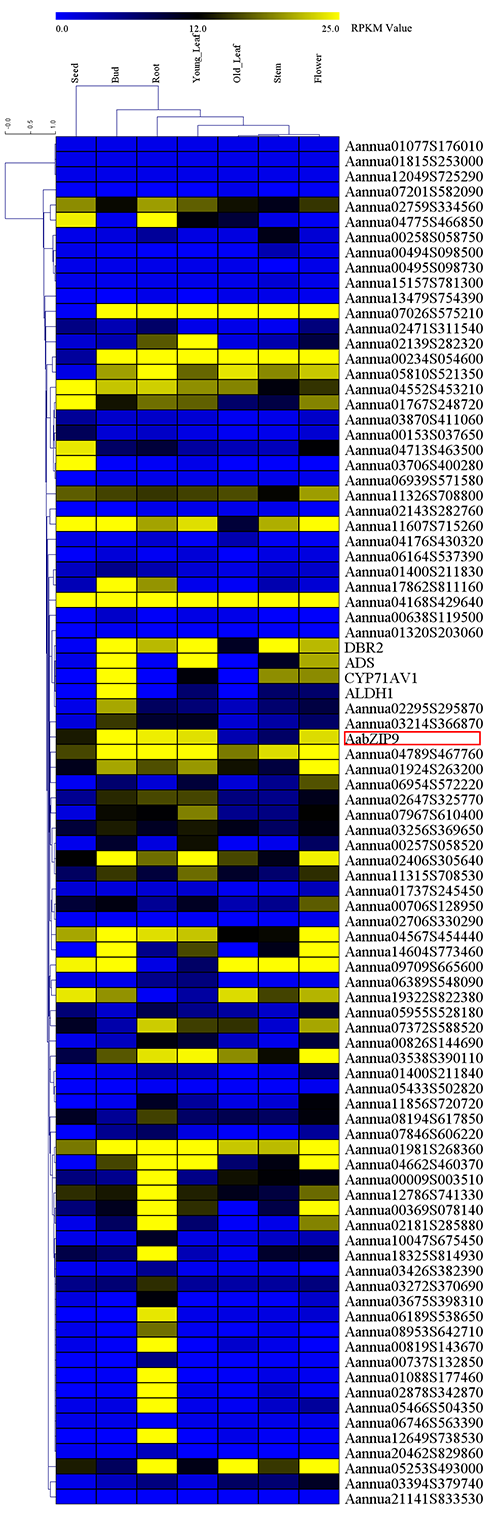

Supplement: Supplementary Figure 1 — Gene co-expression analysis of artemisinin biosynthetic pathway genes with bZIP transcription factor family by MeV 4.9 software. AabZIP9 (marked with red box) gene was clustered with ADS, CYP71AV1 and ALDH1 genes and had a high expression level in the bud, flower, and young leaf, suggesting it may involve in the regulation of artemisinin biosynthesis. [file Image_1.tiff]

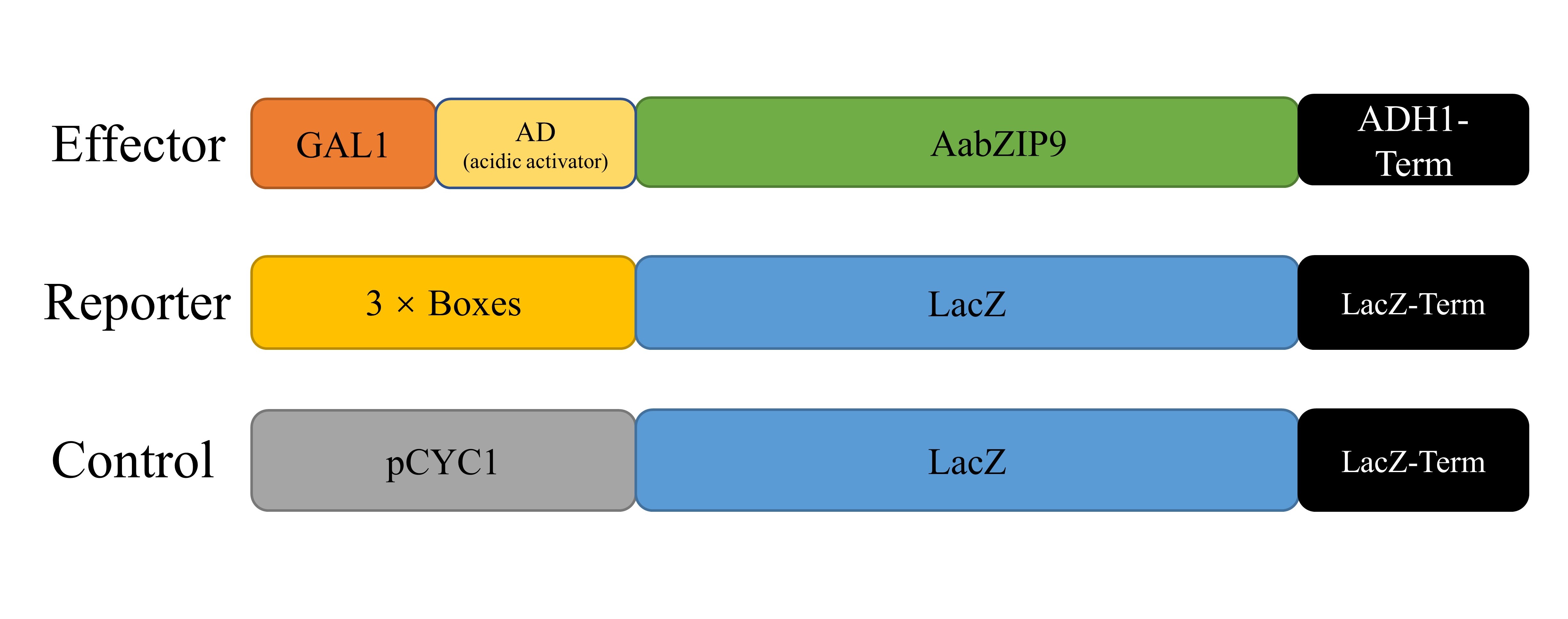

Supplement: Supplementary Figure 2 — Schematic representation of effector and reporter constructs used in the yeast one-hybrid assays. Effector construct contains the AabZIP9 coding sequence driven by the GAL1 promoter while the reporter constructs contain the artificial synthesized triplicate cis-element promoter segments. The empty pLacZ vector was used as control. [file Image_2.jpeg]

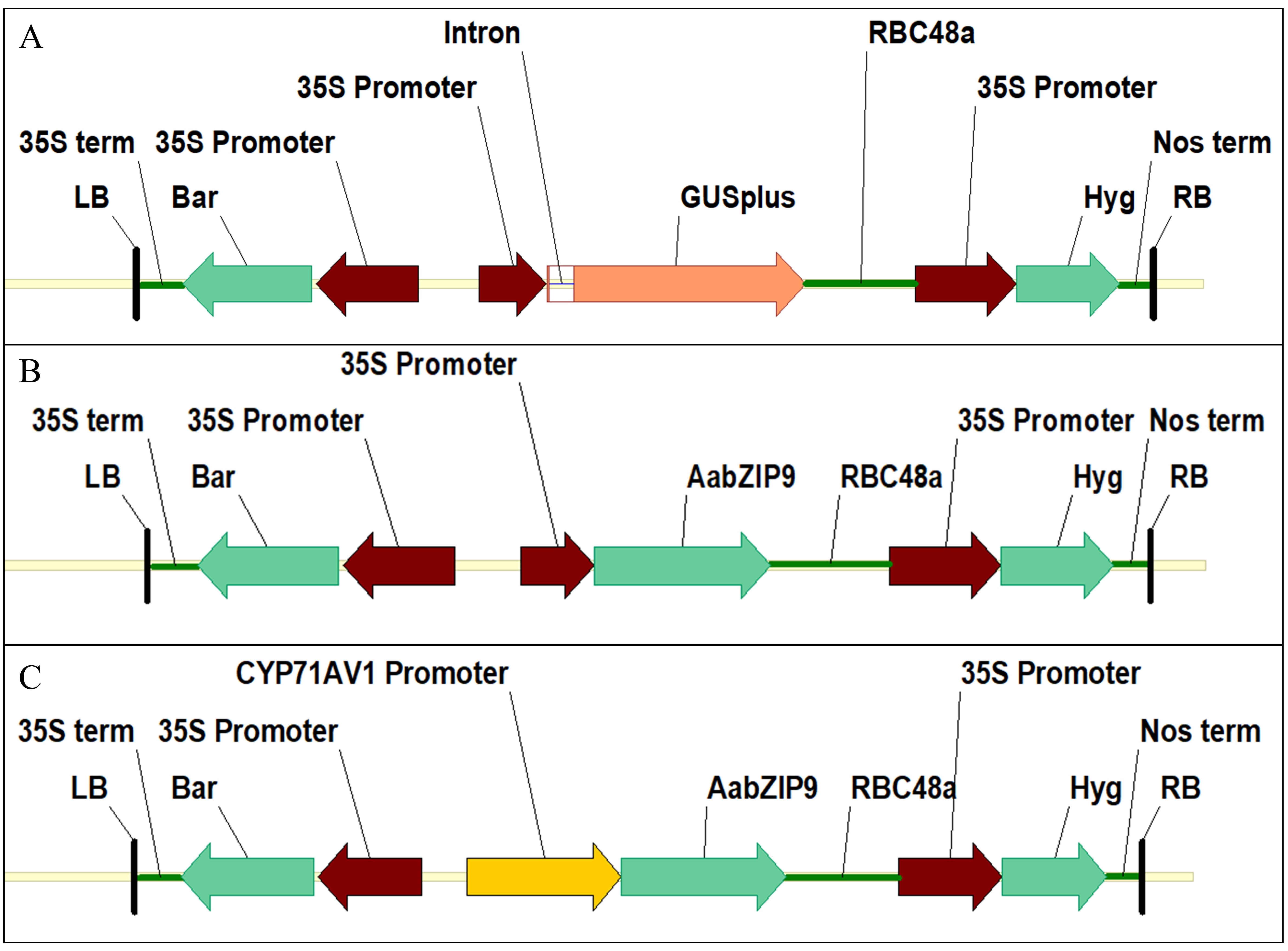

Supplement: Supplementary Figure 3 — Schematic of plant overexpression vector. The AabZIP9 was driven by CaM35S or CYP71AV1 promoter, respectively; Hyg (hygromycin phosphotransferase) was used as resistant selection gene for plant transformation. [file Image_3.jpeg]

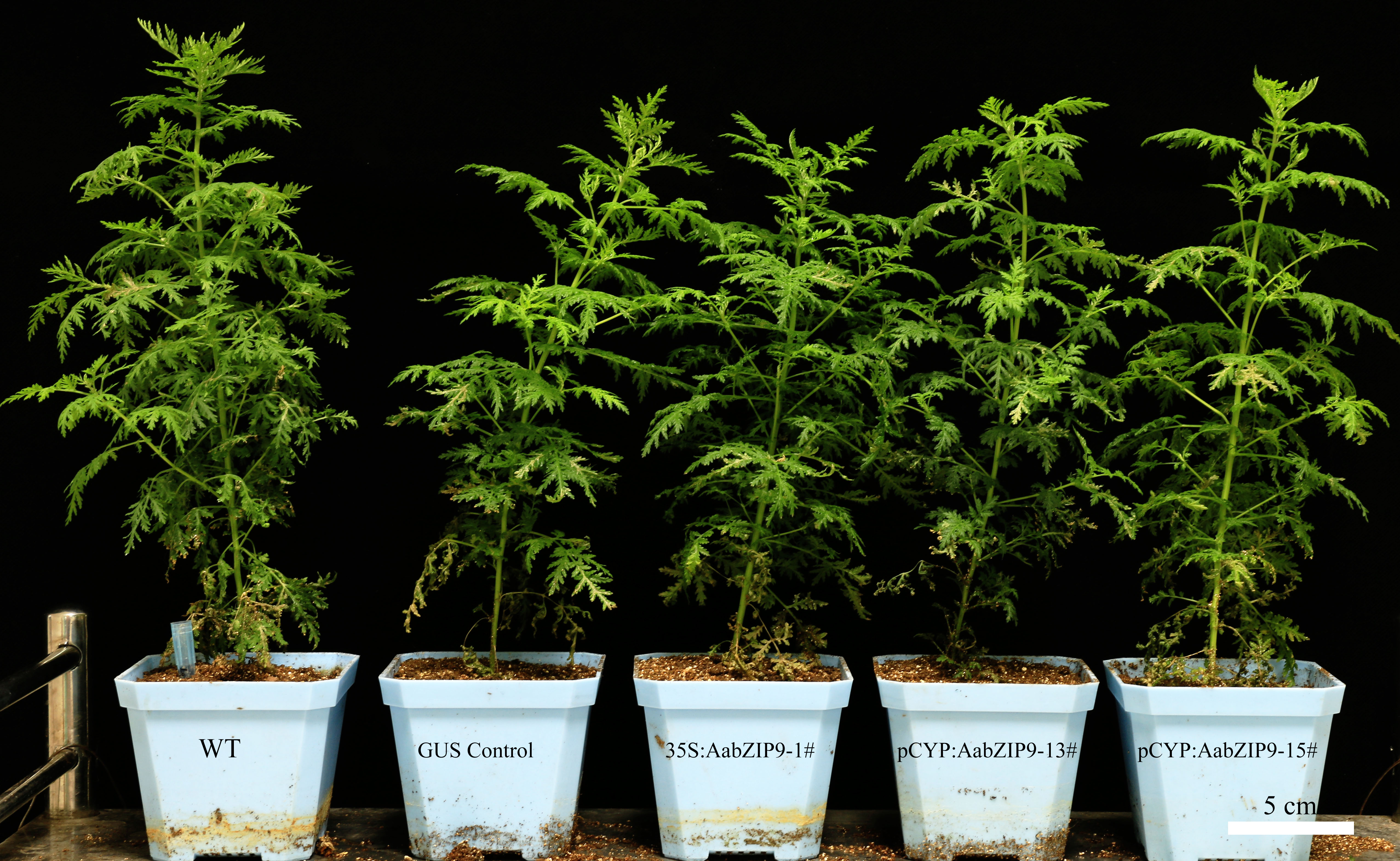

Supplement: Supplementary Figure 4 — The morphologies of 2-month-old wild-type, GUS control, 35S promoter and CYP71AV1 promoter driven overexpressed transgenic plants, and no morphology changed via transformation process; Bar, 5 cm. [file Image_4.jpeg]

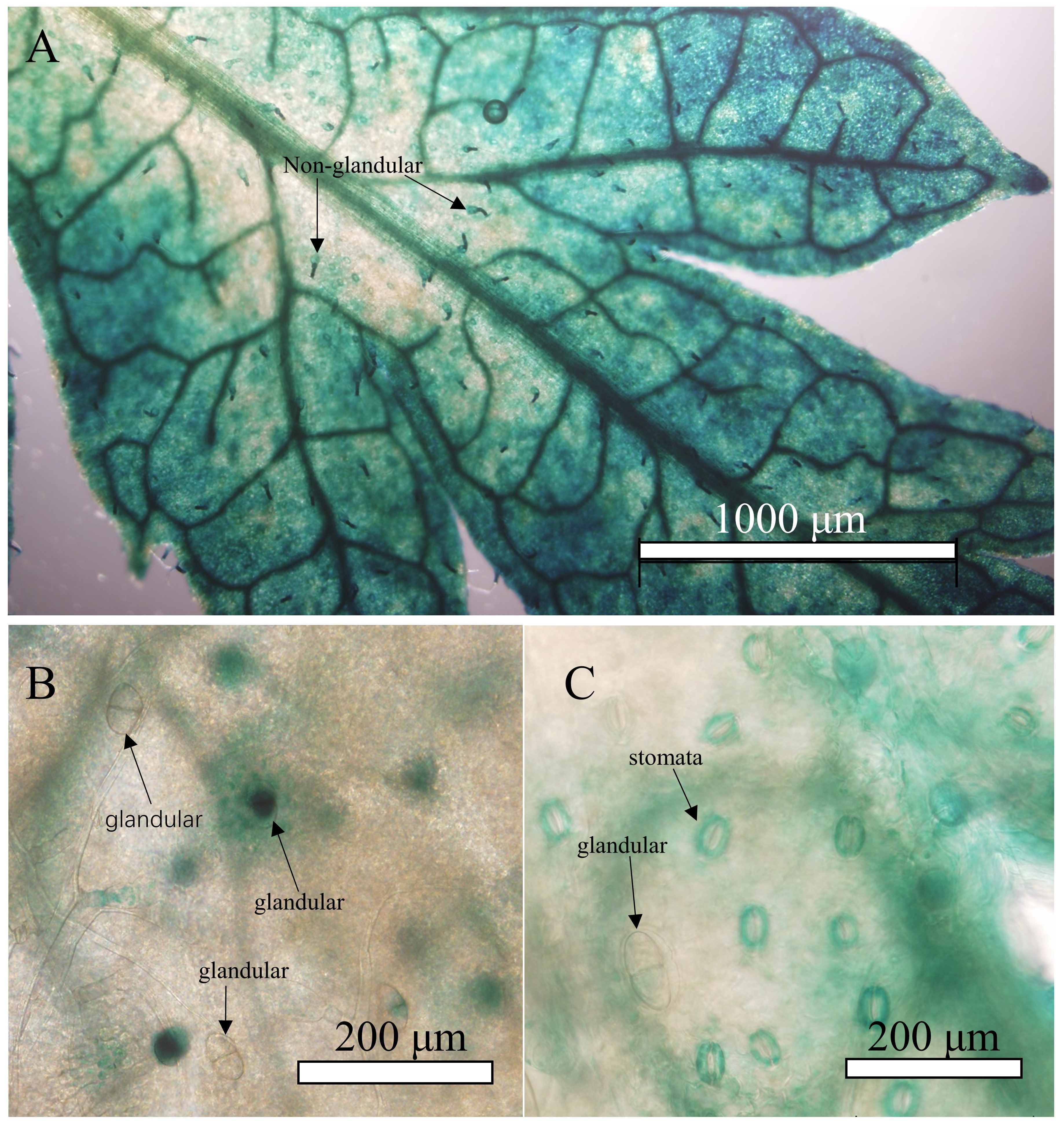

Supplement: Supplementary Figure 5 — Histochemical GUS staining of transgenic A. annua transformed using the 35S: GUS control plasmid. Glandular, nonglandular and stomata structures were pointed out by arrows. (A) 35S promoter has strong activities in most of the leaf tissues (B, C) only a few glandular trichomes get stained by using 35S promoter. [file Image_5.jpeg]
